# Nanomedicines as a cutting-edge solution to combat antimicrobial resistance

**DOI:** 10.1039/d4ra06117a

**Published:** 2024-10-22

**Authors:** Raghu Solanki, Nilesh Makwana, Rahul Kumar, Madhvi Joshi, Ashish Patel, Dhiraj Bhatia, Dipak Kumar Sahoo

**Affiliations:** a Department of Biological Sciences and Engineering, Indian Institute of Technology Gandhinagar Palaj Gujarat 382355 India dhiraj.bhatia@iitgn.ac.in; b School of Life Sciences, Jawaharlal Nehru University New Delhi India; c Dr B. R. A. Institute Rotary Cancer Hospital, All India Institute of Medical Sciences New Delhi India; d Gujarat Biotechnology Research Centre (GBRC) Gandhinagar Gujarat India; e Department of Life Sciences, Hemchandracharya North Gujarat University Patan 384265 Gujarat India uni.ashish@gmail.com; f Department of Veterinary Clinical Sciences, College of Veterinary Medicine, Iowa State University Ames IA USA dsahoo@iastate.edu

## Abstract

Antimicrobial resistance (AMR) poses a critical threat to global public health, necessitating the development of novel strategies. AMR occurs when bacteria, viruses, fungi, and parasites evolve to resist antimicrobial drugs, making infections difficult to treat and increasing the risk of disease spread, severe illness, and death. Over 70% of infection-causing microorganisms are estimated to be resistant to one or several antimicrobial drugs. AMR mechanisms include efflux pumps, target modifications (*e.g.*, mutations in penicillin-binding proteins (PBPs), ribosomal subunits, or DNA gyrase), drug hydrolysis by enzymes (*e.g.*, β-lactamase), and membrane alterations that reduce the antibiotic's binding affinity and entry. Microbes also resist antimicrobials through peptidoglycan precursor modification, ribosomal subunit methylation, and alterations in metabolic enzymes. Rapid development of new strategies is essential to curb the spread of AMR and microbial infections. Nanomedicines, with their small size and unique physicochemical properties, offer a promising solution by overcoming drug resistance mechanisms such as reduced drug uptake, increased efflux, biofilm formation, and intracellular bacterial persistence. They enhance the therapeutic efficacy of antimicrobial agents, reduce toxicity, and tackle microbial resistance effectively. Various nanomaterials, including polymeric-based, lipid-based, metal nanoparticles, carbohydrate-derived, nucleic acid-based, and hydrogels, provide efficient solutions for AMR. This review addresses the epidemiology of microbial resistance, outlines key resistance mechanisms, and explores how nanomedicines overcome these barriers. In conclusion, nanomaterials represent a versatile and powerful approach to combating the current antimicrobial crisis.

## Abstract 2

Antimicrobial mechanism of nanomaterials to combat AMR.

## Graphical abstract

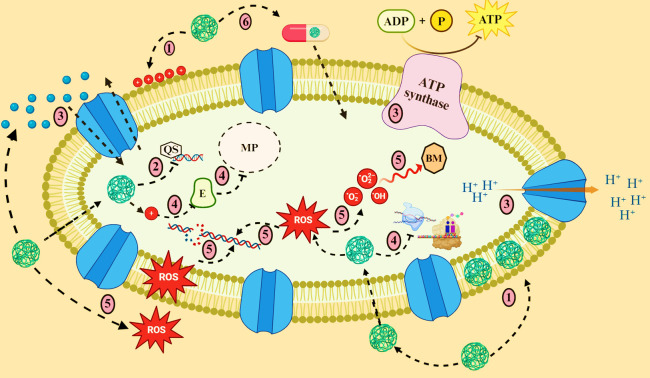

## 1 Introduction

Antimicrobial resistance (AMR) is a growing challenge in modern healthcare, posing a significant threat to progress in the treatment of infectious diseases.^1^ The overuse of antibiotics has led to the rise of drug-resistant pathogens, diminishing the effectiveness of traditional antimicrobial agents. Due to the rapid increase in global resistance, innovative strategies are essential to prevent a potential global healthcare crisis.

Traditional antibiotics are crucial to modern medicine, but they face limitations due to the constant evolution of microbial resistance mechanisms and the slow pace of new antibiotic development.^2^ As a result, there is a widening treatment gap, with once-manageable infections now presenting severe threats. The increasing prevalence of Pan-drug Resistant (PDR) strains further enhances the treatment modality, impacting not only individuals but also healthcare systems, economies, and national security.^3^ Nanomedicines have emerged as a promising solution to combat AMR.^4–6^ Their unique properties enable precise drug delivery, targeted therapies, and immune response modulation, providing a novel avenue to overcome the limitations of traditional antibiotics.^7,8^

Nanomaterials possess novel antimicrobial mechanisms compared to conventional antibiotics. Their small size and high surface-area-to-volume ratio enable multivalent interactions with microbial biomolecules, which can be further enhanced through surface functionalization.^9^ Nanoparticles exhibit high selectivity for microbial cell walls and can penetrate biofilm layers, inducing significant damage to microbial structures.^9^ Additionally, their high surface-to-volume ratio contributes to extended plasma half-lives, facilitating high drug loading and targeted drug delivery. Various metallic nanostructures, such as gold nanoparticles (AuNPs),^10,11^ zinc oxide nanoparticles (ZnO NPs),^12^ silver nanoparticles (AgNPs),^13,14^ and copper oxide nanoparticles (CuO NPs),^15,16^ have been utilized as antimicrobials and nanocarriers for antimicrobial agents, demonstrating significant antimicrobial potency against a wide range of pathogens, including bacteria, viruses, protozoa, and fungi. Metallic nanoparticles generate reactive oxygen species (ROS) and release metal ions, leading to oxidative stress and interference with vital cellular processes.^17,18^ Additionally, nanomaterials can inhibit microbial metabolism, disrupt biofilms, and penetrate intracellularly to target nucleic acids and proteins, thereby enhancing their effectiveness against resistant strains.^19^ Tiwari *et al.* observed that ZnO NPs induced cell wall blebbing and surface irregularities in *Campylobacter jejuni* (*C. jejuni*).^20^ Kadiyala *et al.* also reported that the inhibition of *Staphylococcus aureus* (*S. aureus*) growth was attributed to disruptions in multiple metabolic pathways, including impairments in sugar metabolism and amino acid synthesis by ZnO NPs.^21^ Apart from metallic nanoparticles, polymeric nanocarriers,^4^ lipid,^5^ and polysaccharide-based^22^ have also been used as drug vehicles for antimicrobial agents.

This review aims to explore recent developments in nanomedicine as a potential solution to AMR. It discusses the mechanisms underlying AMR development and how nanomaterials can help combat it, supported by recent studies in the field. By highlighting breakthroughs and innovations with the potential to overcome AMR, the review examines antibiotic resistance mechanisms, recent advancements, and the challenges and opportunities in clinical translation.

## 2 AMR: a global threat

### 2.1 Rise of AMR

**Fig. 1:**
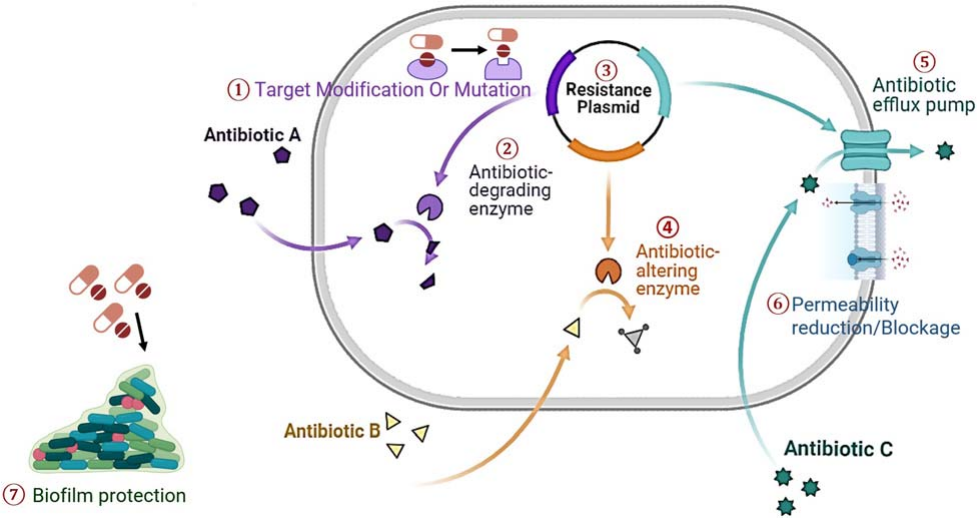
Representations of the mechanism of AMR in microbes.

AMR has evolved as a pressing global health challenge, characterized by the remarkable adaptability of microorganisms, mainly bacteria, in response to the selective pressures of antimicrobial agents.^23^ The advent of antibiotics in the mid-20th century marked a turning point in the medical field, transforming the treatment of microbial infections and significantly extending human life expectancy. However, this success inadvertently resulted in an upsurge of AMR. The irrationality and misuse of antibiotics, often driven by factors such as patient demand, suboptimal prescribing practices, and the widespread use of antibiotics in agriculture, have accelerated the development of drug-resistant pathogens.^24^ Additionally, the high incidence of cross-contamination and medication misuse, which can promote biofilm formation, may lead to nosocomial infections that are resistant to conventional treatment.^25^ AMR can develop through several mechanisms, including the prevention of drug penetration into the cell, alterations in antibiotic targets, enzymatic inactivation of antibiotics, and active efflux of antibiotics from the cell^26^ (Fig. 1). Target modification occurs when mutations in microbial genes alter antimicrobial targets, reducing susceptibility. Pathogens also produce enzymes that degrade or inactivate antibiotics. Horizontal gene transfer allows resistance genes to be transferred between microbes *via* plasmids or other mobile genetic elements.^27^ Additionally, bacteria, viruses, and fungi can employ antibiotic-altering enzymes that modify the antibiotics, making them less effective. Efflux pumps are another mechanism that enables microorganisms to expel antibiotics from their cells actively.^28,29^ Permeability changes, such as cell wall or outer membrane alterations, can reduce antibiotic entry. Biofilm formation provides a protective environment that shields microbes from antibiotics, further complicating treatment. Some pathogenic fungal strains also contribute to biofilm formation and can be more resistant than bacterial biofilms.^30^ These fungal strains are primarily yeasts and filamentous fungi, with *Candida albicans* being the most extensively studied model.^31^

In the context of AMR, the process by which bacteria, viruses, or fungi develop resistance to antimicrobial drugs is a multifaceted phenomenon.^32,33^ Initially, the population of sensitive bacteria, viruses, or fungi characterized by lower resistance to antimicrobial drugs undergoes a decline. Following exposure to antimicrobial agents, these drugs effectively eliminate sensitive microbes, leaving behind a limited number of resistant microbes. As the antimicrobial agents selectively target and kill susceptible strains, the surviving resistant microbes are poised to thrive, particularly when encountering favourable conditions. This selective pressure augments the frequency of resistant strains within the microbes' subpopulation. Furthermore, the transfer of resistance traits plays a crucial role in the spreading of resistance. Resistant pathogens can transfer their resistance traits to nearby microbes, resulting in the contributory spread of antimicrobial resistance within microbial communities. The cumulative consequence of these processes is evident in the transformation of the entire pathogen population. Over time, all microbes within the system undergo a shift, developing resistance to antimicrobial drugs. This intricate interplay of genetic mutations, selective pressure, and horizontal gene transfer underscores the urgent need for comprehensive strategies to mitigate antimicrobial resistance and preserve the efficacy of existing antimicrobial agents. The genetic plasticity of bacteria has enabled them to acquire and disseminate resistance mechanisms swiftly.^34^ Horizontal gene transfer, mutation, and selection pressures have contributed to the emergence of resistance.^35^ Furthermore, the ability of bacteria to develop multidrug resistance, making them impervious to multiple antimicrobial agents, has exacerbated the problem. The limited development of novel antibiotics further aggravates the rise of AMR, as pharmaceutical sectors face diminishing returns on investment in antibiotic research and development.

### 2.2 Consequences of AMR for healthcare

**Fig. 2:**
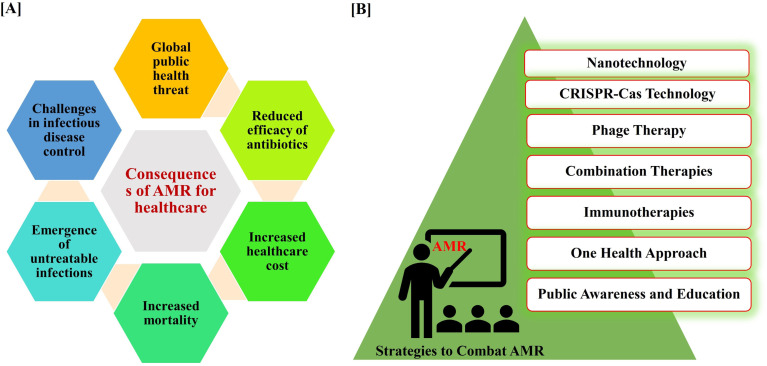
Consequences of AMR in healthcare sectors (A). List of different approaches to combat AMR from nanotechnology to public awareness and education (B).

The consequences of AMR are profound and far-reaching, significantly impacting healthcare systems, economies, and public health^36,37^ (Fig. 2A).

AMR manifests in various detrimental ways, exacerbating mortality rates, economic strains, and healthcare challenges worldwide.^38^ AMR is currently considered to be a chronic public health issue worldwide, with 10 million deaths annually by 2050.^39^ Moreover, AMR imposes a significant economic crisis on healthcare systems, with the United States alone facing an estimated annual cost of $20 billion and $35 billion for lost productivity for treating antibiotic-resistant infections.^38^ Patients afflicted with AMR infections experience prolonged hospitalization, necessitating extended stays and complex treatments that strain healthcare facilities and resources. Additionally, the efficacy of standard medical procedures such as surgery, chemotherapy, and organ transplantation are compromised in the presence of AMR, posing heightened risks to patients. The global spread of AMR further intensifies its impact, transcending borders and posing a substantial threat to global health security. Particularly vulnerable are immunocompromised individuals, such as those with cancer, SARS-Cov-2, or HIV, who face heightened risks due to compromised immune responses and limited treatment options in the face of AMR.^40^

### 2.3 Urgency in finding new strategies to combat AMR

The urgency of finding new strategies to combat AMR cannot be overstated. The World Health Organization (WHO) has identified AMR as one of the foremost worldwide health hazards. The WHO Global Action Plan encourages its member nations to create National Action Plans to address AMR, including measures to rationalize the use of antibiotics in animal health, agriculture, and the food industry.^41^ Since the present strategies for combating AMR lack sufficient multi-sectoral and cross-disciplinary efforts, the “One Health approach” necessitates immediate and synchronized action. The One Health concept promotes initiatives aimed at correcting the inappropriate use of antibiotics in humans, food animals, and the environment.^42^ The current trajectory, if unaltered, could lead to a future where common infections become untreatable, rendering routine medical procedures precarious. It is imperative that we embrace innovative approaches to counteract AMR. Addressing AMR necessitates a comprehensive and varied strategy (Fig. 2B).

Emerging approaches include exploring phage therapy, utilizing CRISPR-Cas technology for gene editing to target antibiotic resistance, implementing antibiotic programs in healthcare, and investigating probiotics and microbiome modulation to enhance natural defences.^43,44^ Additionally, the development of immunotherapies, novel antibiotics, and combination therapies, along with leveraging nanotechnology for targeted drug delivery, are crucial in combatting resistance.^45,46^ Exploring alternatives like antimicrobial peptides and essential oils and prioritizing vaccines against resistant pathogens also plays a key role.^47^ Global surveillance, adopting a One Health approach, and enhancing public awareness contribute to a collective and sustainable effort in the battle against AMR.

Nanomedicines represent a transformative approach in the fight against AMR. They involve the development of nanomaterials to deliver medicines precisely at the target sites in the body. This targeted delivery makes the medication more effective and reduces the risk of bacteria developing resistance. Nanotechnology could be a super-advanced strategy that targets pathogens and improves the efficacy of our medicines.^48^ Therefore, considering this groundbreaking technology to revolutionize and elevate the health system to new heights. In the following sections, we will explore the mechanisms and recent developments in this field, shedding light on its potential to mitigate the consequences of AMR and provide effective strategies for combating this global health crisis.

## 3 Nanotechnology-based strategies

### 3.1 Relevance of nanomedicine in overcoming AMR

**Fig. 3:**
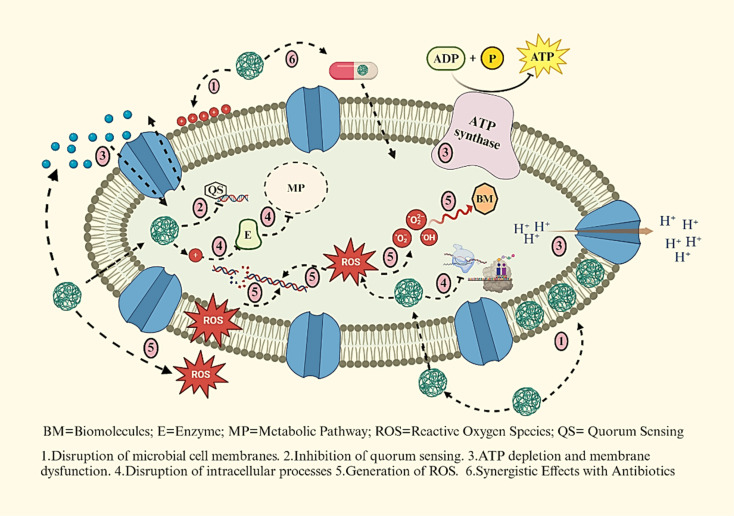
Several mechanisms of nanomaterials to eliminate AMR.

Nanomedicine is an interdisciplinary field that combines nanotechnology, medicine, and biology, aiming to develop, characterize, and apply nanoscale materials and devices in medicinal applications.^49,50^ Nanoscale materials, typically ranging from 1 to 100 nanometers in size, exhibit unique properties that can enhance drug delivery, imaging, and diagnostics.^51^ Nanomaterials have emerged as a promising strategy to combat AMR by employing multiple mechanisms to target microbial cells^52^ (Fig. 3).

Microbial cell walls and membranes play a critical role in AMR, serving as crucial defence barriers.^53^ The structural composition of microorganisms significantly influences their susceptibility to nanomaterials.^54^ For example, nanomaterials are generally more effective against Gram-positive bacteria compared to Gram-negative bacteria. This is because Gram-negative bacteria possess an additional outer membrane rich in lipopolysaccharides (LPS), which forms a robust penetration barrier. In contrast, Gram-positive bacteria have a thinner peptidoglycan layer and a strong negative surface charge, making them more vulnerable to nanoparticle-induced damage.^55,56^ Nanoparticles can penetrate and disrupt bacterial cell walls, leading to membrane damage, ion leakage, ROS formation, and eventual cell death.

Metallic nanoparticles, such as AgNPs,^57^ ZnO NPs,^12^ and AuNPs,^10^ can directly interact with microbial cell membranes, causing destabilization and permeabilization, which leads to leakage of cellular contents and cell death.^58^ The interaction of nanomaterials with bacterial cells could be *via* electrostatic forces, van der Waals interactions, and receptor–ligand binding, subsequently entering and disrupting key metabolic pathways.^59,60^ They also disrupt quorum sensing, a key bacterial communication system that governs biofilm formation and pathogenicity, reducing virulence.^61,62^ Nanoparticles can interfere with bacterial ion gradients by increasing membrane permeability, resulting in disrupted proton motive force and ATP depletion. Furthermore, metal-based NPs inactivate essential enzymes involved in vital metabolic processes, and metal oxide nanoparticles generate ROS that damage lipids, proteins, and nucleic acids. In addition, nanoparticles like ZnO and CuO exhibit synergistic effects when combined with conventional antibiotics, enhancing drug efficacy and reducing bacterial resistance.^63^ Reyes-Torres M. A. *et al.* synthesized metallic nanoparticles (CuO and ZnO) using a green approach.^64^ CuO-NPs were rod-shaped with an average length of 22 nm, while ZnO-NPs were spherical with a mean diameter of 15 nm. Synergy studies revealed that ZnO-NPs showed superior synergistic effects with ampicillin, resulting in a six-fold MIC reduction for most microorganisms except *Pseudomonas aeruginosa*. Together, these mechanisms make nanomaterials a potent alternative to traditional antibiotics for addressing AMR.

The detailed mechanisms of different nanomaterials against pathogens are discussed in Section 3.2. Overall, the application of nanomedicine to combat AMR offers a promising alternative to traditional antibiotics.

### 3.2 Different nanomaterials for antimicrobial therapy

A wide range of nanomaterials, including inorganic and organic nanoparticles, significantly contribute to advancement in antimicrobial healthcare. Nanoparticles can be developed using either a top-down or bottom-up method, with each method offering distinct benefits and applications.^65^ The top-down strategy involves reducing larger materials to nanoparticles using processes like milling or lithography.^66^ In contrast, the bottom-up strategy includes manufacturing nanoparticles from atomic or molecular components, which allows specific control over size, shape, and composition.^67^ While the top-down approach is suitable for mass production and scalability, the bottom-up approach enables fine-tuning of nanoparticle properties for particular purposes including drug delivery, catalysis, or electronics. Understanding and harnessing these synthesis methods are critical for advancing nanotechnology and expanding its diverse range of applications.

**Fig. 4:**
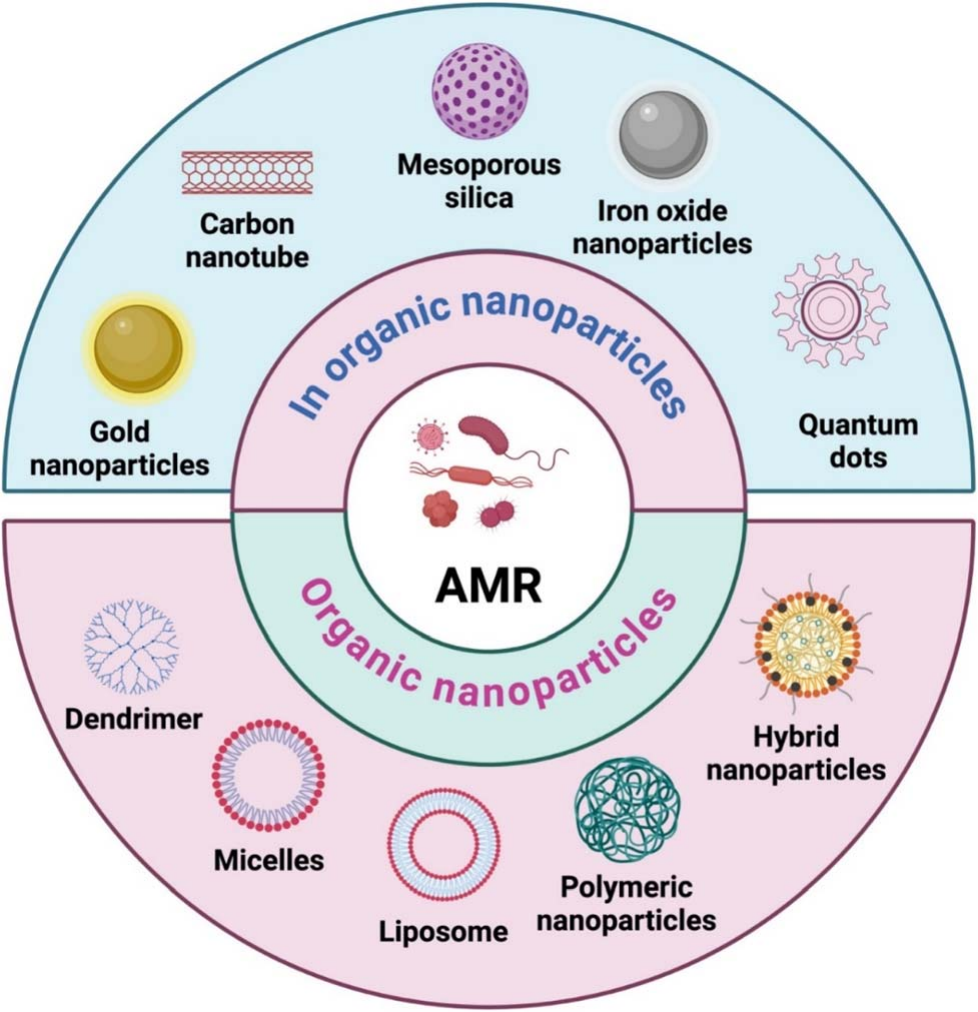
Different nanocarriers used for the delivery of antimicrobial agents (figure prepared using https://Biorender.com).

Inorganic nanomaterials, such as metal nanoparticles (*e.g.*, AgNPs), metal oxide nanoparticles (*e.g.*, ZnO NPs), and quantum dots (QDs), have unique antibacterial characteristics by directly interacting with bacterial structures.^68,69^ On the organic side, polymeric nanomaterials like nanogels and dendrimers provide controlled drug release, improving the stability and efficacy of antimicrobial agents.^70^ Lipid-based nanoparticles, including liposomes, serve as biocompatible carriers for antimicrobial agents.^71^ The versatility of carbon-based nanomaterials, such as carbon nanotubes and graphene, emphasizes their potential for preventing microbial growth. These various inorganic and organic nanomaterials demonstrate the multifaceted and innovative nature of nanotechnology in addressing AMR (Fig. 4).

Several nano-formulations have received FDA approval, with many others in early research or clinical trial phases. The majority of FDA-approved nanomedicines are polymeric- or lipid-based formulations, though clinical trials are also advancing for inorganic and metallic nanoparticles. For example, two FDA-approved polymeric nanomedicines, Pegasys (pegylated IFN alpha-2a) and PegIntron (pegylated IFN alpha-2b), are used to treat hepatitis B and C, respectively.^72,73^ Liposomal formulations, such as the AMB lipid complex and liposomal amphotericin B (AMB), have been approved for treating fungal infections.^74,75^ Doxycycline polymeric nanoparticles (PNs) are currently in Phase II trials for chronic periodontitis and other antimicrobial polymeric nanoformulations, such as EZ and LN, are in Phase I trials for HIV treatment.^76^ Various organic and inorganic nanomaterials used in antimicrobial therapy have been discussed in this review.

### 3.3 Metallic nanomaterials

Metal nanomaterials, such as ZnO NPs, AgNPs, AuNPs, CuNPs, iron oxide nanoparticles (Fe_3_O_4_ NPs) and titanium dioxide nanoparticles (TiO_2_ NPs), are widely used as promising nanocarriers for the antimicrobial drug delivery.^77^ These nanoparticles also used as antimicrobial agents itself. Leveraging their unique properties, including antimicrobial activity, photocatalysis, and magnetic responsiveness, these nanomaterials are being extensively studied for applications in wound dressings, coatings, medical devices, and drug delivery systems.^78^ Furthermore, the antibacterial action of metal nanoparticles is related to their small size, high surface area-to-volume ratio, and ability to release metal ions, which can destroy microbial cells.^79^ AgNPs release silver ions,^80^ AuNPs exhibit optical and catalytic properties,^81^ CuNPs disrupt cell membranes and generate ROS which create additive stress inside the cell leading to cell death,^82^ ZnO NPs demonstrate photocatalytic effects,^83^ TiO_2_ NPs generate ROS when exposed to UV light^84^ and Fe_3_O_4_ NPs possess magnetic properties that enable them to act as antimicrobial agents or deliver antimicrobial agents. Common metallic nanoparticles have been discussed in the following sections.

#### 3.3.1 Silver nanoparticles (AgNPs)

AgNPs are among the most extensively investigated metal nanoparticles due to their effective antimicrobial properties against a variety of pathogens.^57,85^ They are known to be effective against a wide variety of microorganisms, including bacteria, fungi, and certain viruses. The antimicrobial mechanism of AgNPs is multifaceted; they can attach to and penetrate bacterial cell membranes, causing structural changes and leading to cell death. Furthermore, AgNPs can release silver ions (Ag^+^), which add to their antimicrobial activity by interacting with thiol groups in proteins and enzymes, interrupting cellular activities. Rai M. K. *et al.*, have emphasized AgNPs as a strong nano weapon for tackling the AMR.^86^ AgNPs synthesized from *Rubia cordifolia* L. leaf extract^87^ and *Thalictrum foliolosum* leaf extract^88^ demonstrated antifungal activity against *Aspergillus flavus* (*A. flavus*). Bioengineered AgNPs exhibited significant *in vitro* antifungal activity against *A. flavus*, which can be attributed to their optimal particle size and the capping and reducing agents employed during the green synthesis process.

The study done by Roy *et. al.*, demonstrated important role of AgNPs in the fight against antibiotic resistance.^89^ They suggested that AgNPs are highly effective antibacterial agents against a wide range of bacterial strains, including methicillin-resistant *Staphylococcus aureus* (MRSA), vancomycin-resistant *Staphylococcus aureus* (VRSA), erythromycin-resistant *Streptococcus pyogenes*, ampicillin-resistant *Escherichia coli* (*E. coli*), and *Pseudomonas aeruginosa* (*P. aeruginosa*). In another study, AgNPs were synthesized using a green method using the bark extract of *Anadenanthera colubrina* (Vell.) Brenan var. colubrina.^90^ Study demonstrated that prepared AgNPs shown potent antibacterial activity (minimum inhibitory concentration (MIC) = 19.53 − 78.12 μM) against clinical isolates from the ESKAPEE group, a group of bacteria known to cause high hospitalization costs and mortality rates.^85^ This suggests that AgNPs have broad-spectrum antimicrobial potential, which may be essential in addressing the rising issue of antibiotic resistance.

#### 3.3.2 Copper nanoparticles (CuNPs)

CuNPs have demonstrated potential antimicrobial efficacy against a range of microorganism. Like AgNPs, CuNPs have also several ways to produce their antimicrobial effects, such as releasing copper ions that can damage bacterial cell membranes, produce ROS, and interact with DNA.^91^ CuNPs have been found effective against both Gram-positive and Gram-negative bacteria. Salah *et al.*, demonstrated the antimicrobial activity of (CuNP) against different bacterial strains, suggesting their potential as antimicrobial agents in combating drug-resistant bacteria.^92^ Another study done by Hsueh *et al.*, investigated the antimicrobial properties of CuNPs against antibiotic-resistant bacteria, demonstrating their potential as novel antimicrobial agents.^93^

**Fig. 5:**
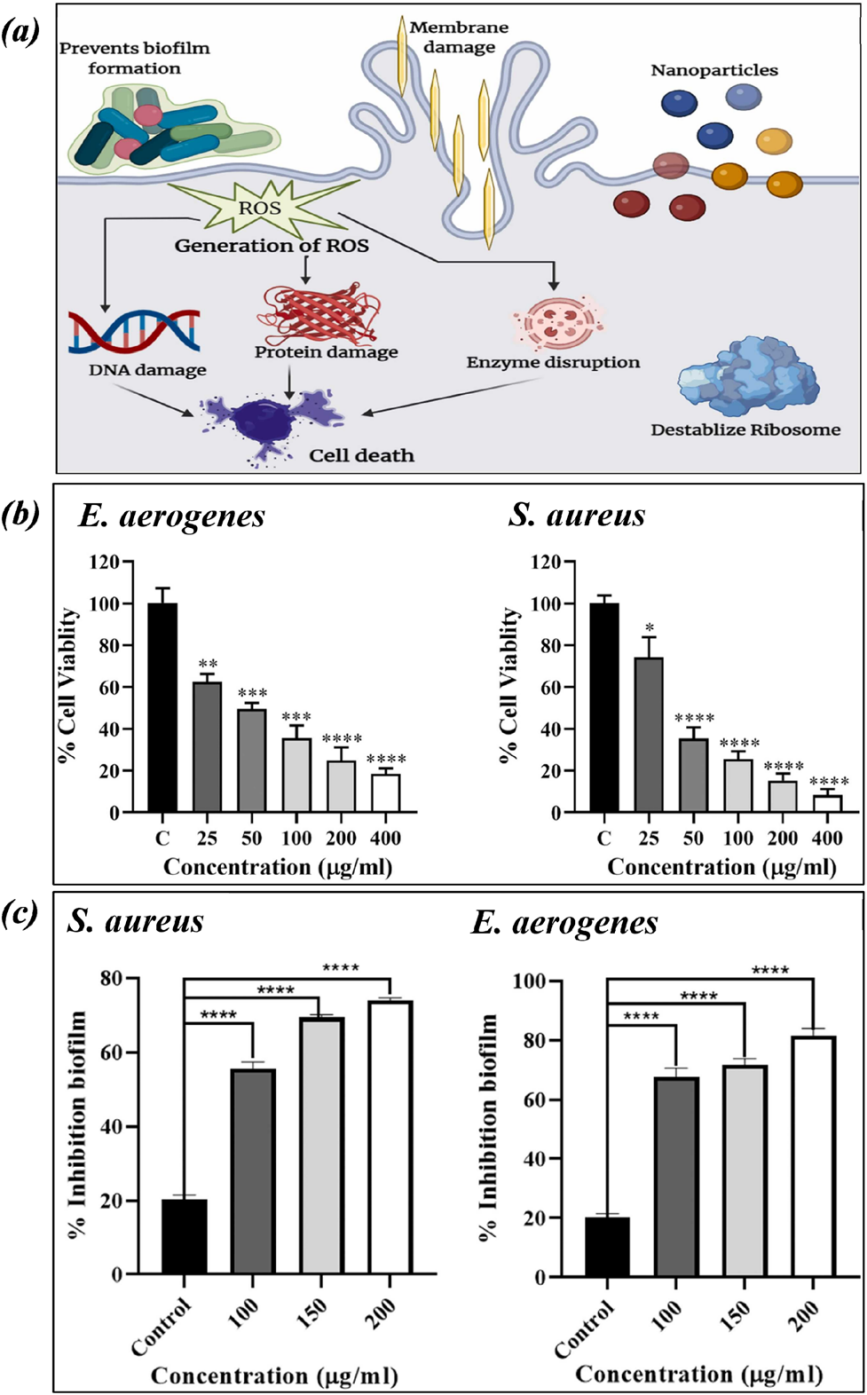
The CuNP-based nanocarrier's mode of action. (a) Zone of inhibition (b) and biofilm reduction (c) of *S. aureus* and *E. aerogenes* after treatment with CuO NS (figures reproduced from ref. 94, Copyright© 2024, Elsevier).

Copper oxide nanosheets (CuO NS) were synthesised from *Celastrus paniculatus* plant extract by Chaudhary, N. *et al.*, and they confirmed the antibacterial activity of CuO NS against strains of *Staphylococcus aureus* (*S. aureus*) and *Enterobacter aerogenes* (*E. aerogenes*).^94^ By demonstrating zone inhibition and biofilm reduction of the specified bacterial strains, the study revealed that synthesised nanosheets have strong antibacterial capabilities (Fig. 5).

Ahamed M. *et al.*, showed antimicrobial activity of CuNPs against *E. coli*, *Klebsiella pneumonia* (*K. pneumonia*), *Salmonella typhimurium* (*S. typhimurium*), *Enterococcus faecalis* (*E. faecalis*), *Proteus vulgaris* (*P. vulgaris*), *S. aureus* and *Pseudomonas aeruginosa* (*P. aeruginosa*). CuNPs shown highest antimicrobial activities against *E. coli* and *E. faecalis* and lower against *K. pneumonia*.^95^ CuNPs have demonstrated a noteworthy reduction of microbial growth in antimicrobial experiments conducted against a range of organisms, such as *E. coli*, *S. aureus*, *A. nigres*, and *C. albicans*. This evidence indicate that CuNPs hold promise as a novel antimicrobial agent with potential applications in the biomedical and pharmaceutical fields.

#### 3.3.3 Zinc oxide nanoparticles (ZnO NPs)

ZnO NPs possess inherent antimicrobial properties including antibacterial, antifungal and antiviral, making them a promising solution for combating AMR.^96,97^ Through a variety of mechanisms, including as the production of ROS, the rupture of microbial cell membranes, and the restriction of microbial growth and biofilm formation, they exhibit antimicrobial action.^98^ ROS such as hydroperoxyl radicals, hydroxyl radicals (OH˙), superoxide ions (O_2_˙−), and H_2_O_2_, generated by ZnO NPs induce lipid peroxidation, cause DNA damage and lead to protein denaturation, ultimately resulting microorganism cell death.^98,99^ Moreover, ZnO NPs exhibit low toxicity to mammalian cells, making them attractive for biomedical applications.^100^ Using *Azadirachta indica* (L.) leaf extract, K. Elumalai and S. Velmurugan synthesised ZnO NPs.^101^ Their antimicrobial activity was tested against strains of bacteria, including *S. aureus*, *Bacillus subtilis* (*B. subtilis*), *P. aeruginosa*, *Proteus mirabilis* (*P. mirabilis*), and *E. coli*, and strains of fungi, including *Candida albicans* (*C. albicans*) and *Candida tropicalis* (*C. tropicalis*). The study shown significant antimicrobial activity and it was increased with increasing concentrations (50, 100, and 200 μg mL^−1^) of ZnO NPs due to increased H_2_O_2_ concentration on the surface of ZnO. In another study done by Reddy S.L. *et al.*, demonstrated that ZnO NPs plays a unique antibacterial role in *K. pneumonia* by disrupting the bacterial cell wall membrane and binding to intracellular substances.^102^ The results of the experiments demonstrate ZnO NPs as a potentially strong bactericidal and antifungal properties.

#### 3.3.4 Gold nanoparticles (AuNPs)

AuNPs have emerged as a versatile platform in the fight against due to their unique properties, including high surface area-to-volume ratio, tunable size, and ease of functionalization.^103^ Numerous studies have explored antimicrobial properties of AuNPs conjugated/loaded with self-assembled peptides,^104^ antimicrobial drugs,^105^ vaccines^106^ and antibodies,^107^ demonstrating their potential in combating AMR. Their intrinsic antibacterial, antifungal, and antiviral activities make them a promising alternative to traditional antibiotics. Several research groups have successfully synthesized AuNPs using extracts from organisms such as bacteria, fungi, and plants using biosynthesis and bioengineering approaches.^108,109^

The antimicrobial efficacy of AuNPs has been extensively studied against various pathogens, including Gram-positive and Gram-negative bacteria, as well as fungal strains. Research indicates that AuNPs can penetrate bacterial cell walls and membranes, disrupting vital cellular functions and ultimately leading to cell lysis.^110^ For example, Rajchakit *et al.*, synthesized antimicrobial peptide (AMP)-conjugated AuNPs with a controlled size of 10 nm and evaluated their antibacterial properties.^111^ The AMP-conjugated AuNPs demonstrated potent antimicrobial activity against clinical isolates, including *S. aureus*, *P. aeruginosa*, and *Acinetobacter baumannii* (*A. baumannii*). Additionally, the conjugates exhibited promising anti-biofilm activity and were stable in serum, showing low toxicity *in vitro* and *in vivo*. Furthermore, the incorporation of antimicrobial drug within AuNPs enhances their antimicrobial potency. The reported study suggests that AuNPs enhance the antibacterial activity of ciprofloxacin (CIP) against *E. coli* and *S. aureus*, demonstrating the potential of sonodynamic antimicrobial chemotherapy.^105^

**Fig. 6:**
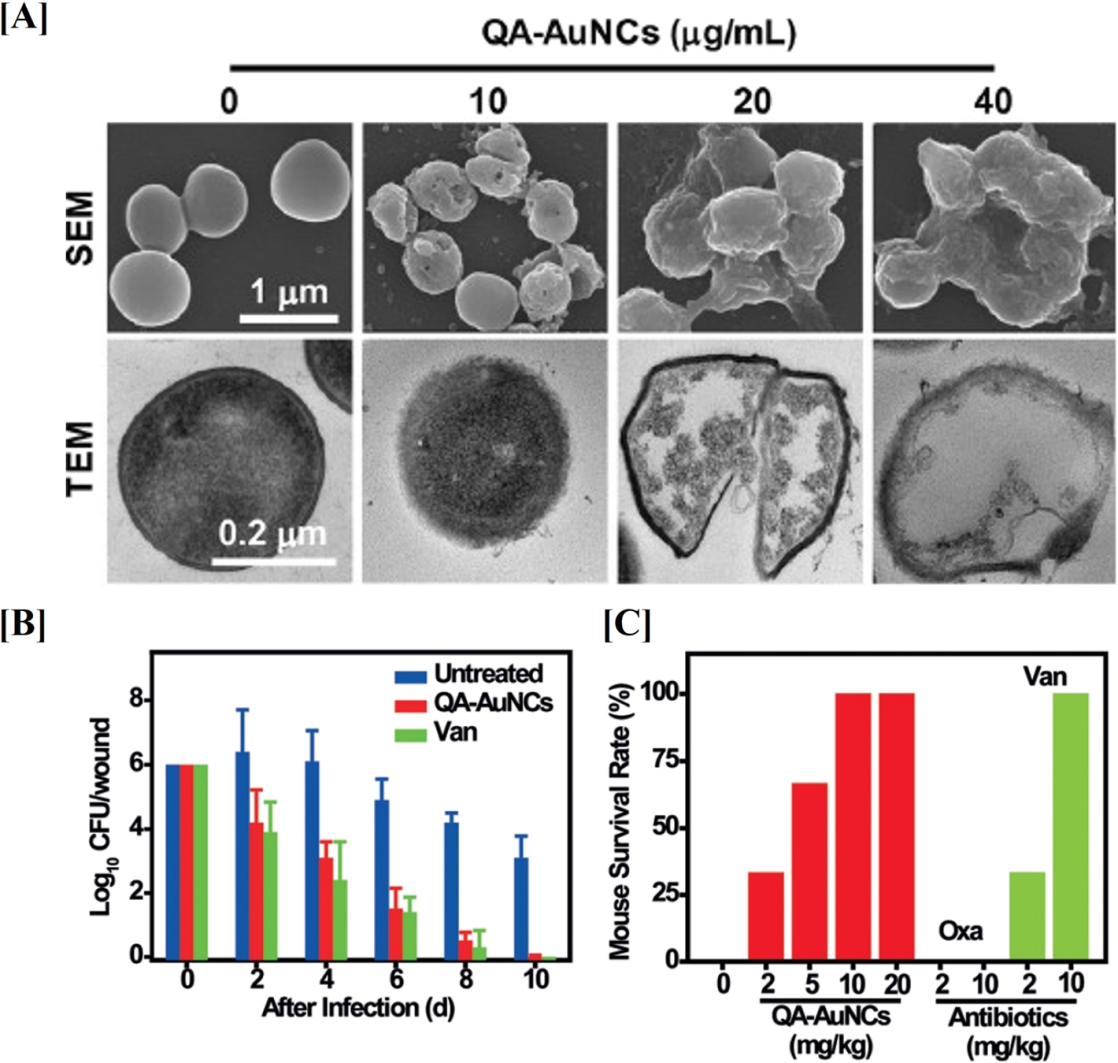
Gold nanostructures for targeting *S. aureus*. SEM and TEM images of gold nanostructures treated *S. aureus* (A). Therapeutic effects of gold nanostructures on MRSA infections *in vivo*, reducing bacterial counts in a mouse skin infection model (B) and improving survival rates in a bacteremia model (C) when combined with oxacillin and vancomycin (figures reproduced from ref. 112 with permission, Copyrights© 2018 Wiley).

Xie Y. *et al.*, developed gold nanoclusters (AuNCs) to combat multidrug-resistant (MDR) bacteria *in vivo*.^112^ In their study, they synthesized Quaternary ammonium capped AuNCs (QA-AuNCs), a novel type of AuNC designed to specifically target MDR Gram-positive bacteria, including MRSA and Vancomycin-Resistant *Enterococci* (VRE). Developed QA-AuNCs eliminate bacteria through a combined physicochemical mechanism and demonstrated significant therapeutic efficacy in both a skin infection model and a MRSA-induced bacteremia model (Fig. 6). In conclusion, the evidence on AuNPs demonstrates their therapeutic potential and promising role in combating AMR. These studies suggest that AuNPs, when combined with antibiotics or antimicrobial peptides, significantly enhance bacterial targeting, biofilm disruption, and *in vivo* therapeutic efficacy against MDR pathogens such as MRSA and VRE.

### 3.4 Lipid based nanocarriers

Lipid-based nanocarrier's amphiphilic nature offers several advantages, including the ability to encapsulate both hydrophilic and hydrophobic antimicrobial compounds.^113^ Liposomes, solid lipid nanoparticles (SLNs), and nanostructured lipid carriers (NLCs) are among the types of lipid-nanocarriers that have attracted a lot of attention as versatile delivery systems for antimicrobial agents.^114^ These nanocarriers offer advantages such as biocompatibility, controlled release, and the ability to encapsulate both hydrophobic and hydrophilic drugs.^115^ Liposomes, composed of lipid bilayers, can encapsulate antimicrobial agents within their aqueous cores or lipid membranes. Solid lipid nanoparticles and nanostructured lipid carriers, on the other hand, provide stable matrices for drug encapsulation. These lipid nanocarriers can improve the solubility and bioavailability of antimicrobial agents and enable targeted delivery to infection sites. Moreover, their surface can be modified for enhanced stability and specific targeting. Lipid nanocarriers hold promise for applications in various medical contexts, including topical formulations, intravenous delivery, and localized treatments, contributing to the development of effective and tailored antimicrobial therapies.^116^ The clinical approval of Doxil in 1995 was a significant milestone for cancer nanomedicine and lipid-based drug delivery systems.^117^ Since then, various lipid based nanocarriers have been synthesized for the delivery of antimicrobial agents, chemotherapeutic drugs, nutrients, phytochemicals and so on.^118^

The toxicity of polymyxin B limits its systemic use even if it is efficient against Gram-negative bacteria. After encapsulation in liposomes, the antibacterial activity was increased with lowering toxicity.^119^ Severino P. *et al.*, also synthesized SLNs for the same drug using the water-in-oil-in-water (w/o/w) emulsion method.^120^ The Polymyxin loaded SLNs exhibited inhibition of *P. aeruginosa* suggests the antibacterial activity of SLNs. Nisin possesses antimicrobial and anti-biofilm properties against both Gram-positive and Gram-negative drug-resistant pathogens. It is commonly used as a preservative in heat-treated and low-pH foods, though it can lose its bioactivity upon contact with dietary components. To overcome this limitation, Prombutara P. *et al.*, developed nisin-loaded SLNs using high-pressure homogenization.^121^ These SLNs protect nisin from degradation in the food environment and extend its biological activity. Nisin-loaded SLNs demonstrated sustained antibacterial efficacy against *Listeria monocytogenes* DMST 2871 and *Lactobacillus plantarum* TISTR 850, outperforming free nisin.

**Fig. 7:**
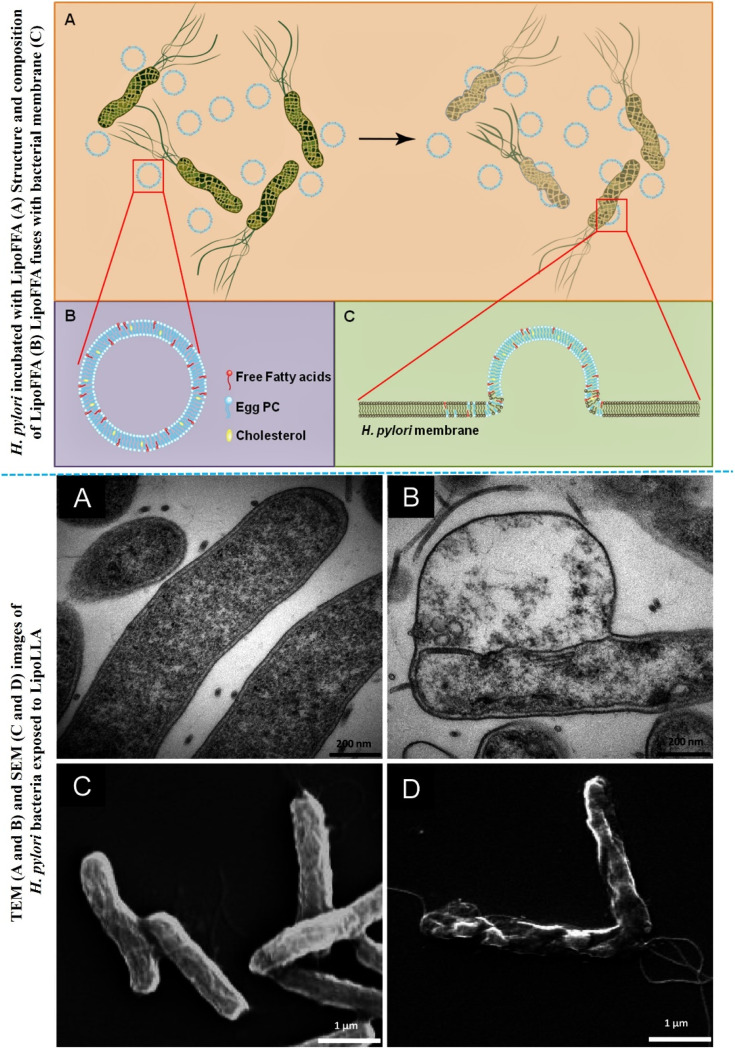
Schematic representation of structure of LipoFFA and its incubation with *H. Pylori* (upper figure). TEM and SEM images of *H. Pylori* exposed with LipoLLA (lower figures) (figures reproduced from ref. 122, Copyright© 2015 Jung *et al.*, PLOS One).

*Helicobacter pylori* (*H. pylori*) infect the human stomach lining, leading to conditions like ulceric cancer. Antimicrobial-loaded liposomes offer a promising approach to effectively combat *H. pylori* infections, potentially reducing the risk of associated cancer. Jung, S. W., *et al.*, developed a lipid-based nanocarrier (liposomal linoleic acid, LipoLLA) for antibacterial effect against *H. pylori*.^122^ The results from *in vitro* and *in vivo* studies suggest promising antibacterial efficiency of LipoLLA against *H. pylori*. Within five minutes, LipoLLA disrupted the bacterial membrane's structure, compromising its integrity and causing cytoplasmic contents leakage, as confirmed by TEM and SEM analysis (Fig. 7). These results demonstrate the rapid bactericidal action of LipoLLA, implying its potential as a highly effective new anti- *H. pylori* medicine.

Lipid-based nanomaterials are regarded as safe nanocarriers due to their nanoscale size, high stability, low or minimum toxicity and controlled drug release properties. When loaded with antimicrobial drugs, these nanocarriers significantly enhance antimicrobial activity compared to free agents, enabling the use of lower drug concentrations.

### 3.5 Micelles

Micelles, as nanocarriers for antimicrobial agents, present a promising strategy due to their unique amphiphilic structure, consisting of hydrophobic cores and hydrophilic shells.^123^ These self-assembling structures enable the encapsulation of hydrophobic antimicrobial compounds within the core, enhancing their solubility and stability.^124^ Micelles, often composed of surfactant molecules, facilitate improved drug delivery and release profiles. The hydrophilic shell allows for interactions with biological environments, making them suitable for various administration routes. Their nanoscale size and ability to incorporate diverse antimicrobial agents contribute to their versatility. Micellar drug delivery systems have demonstrated promise in enhancing the efficacy of antimicrobial treatments, include overcoming drug resistance issues.^125^

Popovici C. *et al.*, synthesized micelles using Pluronic F127 and antimicrobial properties were assessed against Gram-positive and Gram-negative bacteria.^126^*In vitro* biological experiments revealed the hemocompatible and cytocompatible nature of micelles and exhibited increased inhibition zones of 36 and 20 mm against *S. aureus* and *E. coli*, respectively. In another study, Yang S. C. *et al.*, designed Soyaethyl morpholinium ethosulfate (SME) micelles and their antibacterial activity was evaluated against *S. aureus* and methicillin-resistant *S. aureus* (MRSA).^127^ The minimum inhibitory concentration 1.71 to 3.42 and 1.71 to 6.84 μg mL^−1^ were observed for *S. aureus* and MRSA, respectively. SME micelles also showed significant antimicrobial effects in mice model and decreased cutaneous infection and MRSA load. In conclusion, micelle-based nano-drug delivery systems uniquely address the challenges of antimicrobial therapies by offering dynamic structural adaptability and efficient drug encapsulation. Their ability to penetrate biofilms and deliver antimicrobial agents precisely at infection sites makes them particularly effective against resistant pathogens, presenting a novel strategy in combating AMR.

### 3.6 Dendrimers

Tomalia and his colleagues systematically attached branches to a central core molecule, mimicking the hierarchical structure of trees to regulate molecular growth in an orderly manner (dendra is the Greek word for tree).^128^ Dendrimers, three-dimensional nanopolymeric structures with well-defined branches, have emerged as an efficient class of nanocarriers for antimicrobial medicines.^129^ Their unique architecture allows precise control over size, shape, and surface functionalities, offering advantages in drug delivery applications. Dendrimers can encapsulate antimicrobial compounds within their interior cavities or covalently attach them to their functional groups on the periphery.^130^ This controlled drug loading capability enhances solubility and bioavailability of antimicrobial compounds. The multivalency of dendrimers enables interactions with microbial surfaces, enhancing targeting and antimicrobial efficacy. Furthermore, dendrimers have shown potential in disrupting bacterial biofilms and overcoming drug resistance mechanisms.^131^

Different types of dendrimers, specifically cationic polyamidoamine (PAMAM) dendrimers, were explored as carriers for conventional antibiotics to enhance their antimicrobial efficacy. The antibacterial activity of modified polycationic and polyanionic dendrimers in combination with levofloxacin (LVFX) was examined by Wrońska N. *et al.*, against Gram-positive (*e.g.*, *S. aureus*) and Gram-negative (*e.g.*, *E. coli* and *P. hauseri*) bacteria.^132^ Due to the synergistic effects of dendrimer and LVFX, enhanced antibacterial activity was observed. The electrostatic attraction between the positive charges of the dendrimer and the negative charges of the bacterial surface, the progressive permeabilization of bacterial membranes, and the disruption of the lipid bilayer are some of the factors or physicochemical properties that affect the effectiveness of antimicrobial action in dendrimers.^133^ Serri A. *et al.*, evaluated the impact of generations 3 and 5 polyamidoamine amine-terminated dendrimers (NH2-PAMAM) on the antibacterial activity of vancomycin against *E. Coli*, *K. pneumonia*, *S. typhimurium*, and *P. aeruginosa*.^134^ The findings showed that while the vancomycin solution responded effectively against Gram-positive bacteria, it was ineffective against Gram-negative bacteria. Gram-negative bacteria were significantly inhibited by vancomycin-PAMAM dendrimers, which reduced the vancomycin MIC values by around 2, 2, 4, and 64 times in *E. Coli*, *K. pneumonia*, *S. typhimurium*, and *P. aeruginosa*, respectively. Furthermore, the results revealed that G5 has a greater enhanced effect than G3. These findings using dendrimers are promising for increasing the antibacterial range of antimicrobial agents like vancomycin and LVFX.

### 3.7 Polymeric nanocarriers

Polymeric nanocarriers have emerged as versatile platforms for delivering antimicrobial agents, offering unique properties such as biocompatibility, tunable properties, and sustained release profiles.^135^ These nanocarriers are made of biodegradable and biocompatible polymers such as PEG, albumins, chitosan, PLGA and so on.^136^ Surface modifications can facilitate targeted delivery to specific sites of infection, and stimuli-responsive polymers enable triggered drug release in response to physiological cues. Polymeric nanocarriers have shown promise in addressing challenges like drug stability, improving drug pharmacokinetics, and overcoming microbial resistance.^137^

Geraniol is a component found in rose oil that has the suppressive ability to the growth of numerous foodborne bacterial and fungal pathogens. Further to enhance the antimicrobial properties of Geraniol, Yegin Y. *et al.*, designed Pluronic F127 based polymeric nanoparticles.^138^ Geraniol-loaded NPs showed sustained release with a time constant of 24 hours, indicating that their anti-pathogenic capabilities may be maintained over time. Geraniol nano-encapsulation boosted antibacterial action against the pathogens *S. Typhimurium* and *E. coli* O157:H7 by reducing the quantity of Geraniol required for inhibition due to improved Geraniol delivery to pathogen membranes. Another study conducted by Andriotis E. G. *et al.*, also demonstrated the use of polymeric nanoparticles as a drug delivery vehicle for d-limonene, the main ingredient of citrus essential oils.^139^ The antibacterial properties of the synthesised polymeric nanoparticles were evaluated against four microorganisms. The efficacy of the loaded d-limonene exhibited potential antibacterial activity. Additionally, the addition of ε-polylysine to the d-limonene nano-emulsion enhanced its antibacterial efficacy. Overall, the results confirm that d-limonene nano-emulsion has antibacterial properties even at low concentrations.

**Fig. 8:**
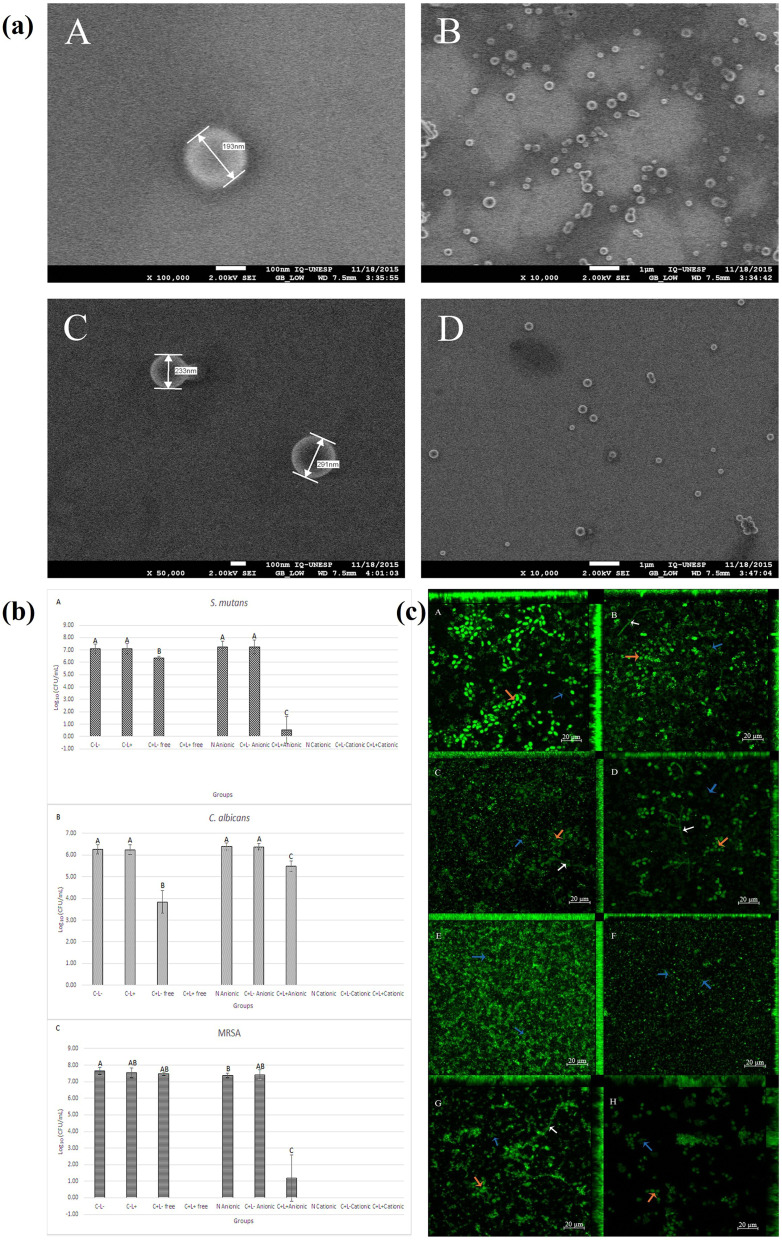
FEG-SEM images of CUR loaded polymeric nanoparticles (a). Mean values of log_10_ (CFU mL^−1^) of planktonic culture (b) and uptake of CUR loaded polymeric nanoparticles by the triple species biofilms (c) (figures reprinted with permission from ref. 140, Copyright© 2017).

Trigo Gutierrez J.K. *et al.*, designed Curcumin (CUR) loaded polymeric nanoparticles (CUR-NPs) by nanoprecipitation approach using polylactic acid and dextran sulfate.^140^ The antimicrobial activities of prepared CUR-NPs were assessed against different bacterial strains including *S. mutant*, *C. albicans* and MRSA. The synthesized CUR-NPs are spherical and nanoscale sized (193 and 214 nm), improved aqueous solubility, enhanced antimicrobial activities against planktonic cultures and significant reduction of biofilms (Fig. 8).

### 3.8 Mesoporous nanomaterials

Mesoporous nanomaterials, characterized by their well-ordered and interconnected porous structures with pore sizes typically in the mesoporous range (2–50 nm), have gained considerable interest in the field of antimicrobial delivery.^141,142^ These materials composed of silica, metal oxides, or polymers, offer a high surface area and controllable pore size, providing an ideal environment for loading and releasing antimicrobial agents.^143^ Mesoporous silica nanoparticles (MSNs), for instance, can encapsulate a variety of drugs within their pores, allowing for sustained and controlled release.^144^ The unique structural characteristics of mesoporous nanomaterials facilitate the protection of encapsulated antimicrobial agents from degradation and enhance their bioavailability. Moreover, surface modifications can be employed to achieve targeted drug delivery and improved biocompatibility. As a result, mesoporous nanomaterials represent a promising avenue for addressing challenges in antimicrobial therapy.

Cadena M. B. *et al.*, encapsulated essential oils into MSNs to extend and improve their antibacterial action resulted in a 10-fold increase in potency when compared to free essential oils.^145^ Tian Y. *et al.*, decorated MSNs with AgNPs and confirmed the antimicrobial activity of these Ag-MSNs against both Gram-positive and Gram-negative bacteria.^146^ The enhancement could be attributed to MSNs preventing silver nanoparticle agglomeration and continuously releasing silver ions over one month. In another study done by de Juan Mora B. *et al.*,^147^ synthesized two novel MSNs based nanomaterials MSN-[Ch][Cip] and MSN-Cip, both incorporating ciprofloxacin within MSNs. Both materials showed promising antimicrobial activity against Gram-positive and Gram-negative bacteria, outperforming the precursor [Ch][Cip] and free ciprofloxacin. MSNs were also conjugated with an FB11 antibody targeting lipopolysaccharide and model drugs fluorescein and Hoechst 33342 were loaded to target *F. tularensis*.^148^ These studies confirmed the promising use of MSNs to in the treatment of bacterial treatment.

### 3.9 Hydrogels

Hydrogels have emerged as versatile and promising carriers for antimicrobial agents, offering a three-dimensional, water-swollen network that mimics the natural extracellular matrix.^149,150^ These materials, composed of polymers like polyacrylates, polyethylene glycol, or natural polymers such as alginate or chitosan, provide a conducive environment for loading and delivering antimicrobial compounds.^151^ Hydrogels can absorb and retain a significant amount of water, facilitating the controlled release of encapsulated antimicrobials. Their high-water content also enhances biocompatibility and supports tissue-like properties, making them suitable for biomedical applications. Antimicrobial-loaded hydrogels have been reported for applications in wound dressings, coatings, and tissue engineering, offering sustained drug release to effectively combat infections.^152^ Additionally, the tunable mechanical and structural properties of hydrogels allow for tailoring drug release kinetics and optimizing therapeutic outcomes.^153^ Ongoing research is focused on developing smart, stimuli-responsive hydrogels capable of releasing antimicrobial agents in response to specific triggers, such as pH or temperature changes.^150,154^ This innovation significantly enhances their efficacy in targeted applications and represents a significant advancement in the field of controlled antimicrobial delivery.

Amphotericin B is well known for its broad antifungal spectrum and is used to combat medical device-related infections. Zumbuehl A. *et al.*, investigated dextran based antifungal hydrogels for the absorption of Amphotericin B.^155^ The resulting formulation (Amphogel), exhibited rapid fungicidal activity within two hours of contact and retained potency against *C. albicans* for at least 53 days. It demonstrated biocompatibility and did not induce hemolysis in human blood. *In vivo* studies revealed its efficacy in protecting against fungal infections and reducing biofilm formation when implanted in mice. De Giglio E. and colleagues investigated new, promising, and adaptable materials made of thin films of either poly(2-hydroxyethyl methacrylate) or a copolymer based on poly (ethylene–glycol diacrylate) and acrylic acid.^156^ These polymers electro-synthesized directly onto titanium substrates and then loaded with CIP. The *in vitro* inhibitory effect of this hydrogel formulation was assessed on the growth of MRSA. MG63 human osteoblast-like cells were then used to evaluate hydrogel coatings for biocompatibility. The findings indicate that antibiotic-modified hydrogel coatings could be a viable way to prevent dangerous bacterial infections commonly associated with orthopaedic surgery without impairing osteoblast processes related to new bone formation.

### 3.10 Nanofibers

Nanofibers have gained prominence as effective carriers for antimicrobial agents, offering a unique combination of high surface area, porosity, and tunable properties.^157^ Nanofibers can be fabricated through techniques like electrospinning, phase separation and self-assembly. They can be composed of various materials, including polymers such as poly(lactic acid) (PLA), poly(ethylene oxide) (PEO), or blends with natural polymers like chitosan or gelatin.^158^ The electrospinning process allows for the production of nanofibers with diameters in the nanoscale range, resembling the fibrous structure of the extracellular matrix.^159,160^ Antimicrobial agents can be incorporated into nanofibers during the fabrication process, leading to controlled release profiles. Nanofiber-based antimicrobial systems find applications in wound dressings, air filtration, and tissue engineering, where the fibrous structure promotes cell adhesion and proliferation while simultaneously preventing microbial infections.^161^ In research conducted by Chan W. P. *et al.*, a nonwoven mat using Silk Fibroin Protein (SFP) blended with baicalein (BAI) was prepared.^162^ The designed SFP/BAI and SFP/PVP/BAI nonwoven mats are engineered by an electrospinning approach. The *in vivo* findings demonstrated that in mice treated with SFP/PVP/BAI nonwoven mat, the wound closure process is significantly accelerated, there is less neutrophil infiltration, nitrite production, and wound bacteria development are inhibited. In another study, Yao C. *et al.*, fabricated gelatin nanofibers containing *Centella asiatica* extract using the electrospinning method.^163^ The prepared nanofibers demonstrated significant wound-healing activity in a rat model. Rats treated with gelatin nanofibers containing *C. asiatica* showed the highest wound recovery rate compared to those treated with gauze, neat gelatin membranes, and commercial wound dressing. These findings highlight the potential nanofibers in skin wounds and antimicrobial infections.

Significant results demonstrating the antibacterial action of various nanoparticles are summarized in Table 1. This table includes the type of nanoparticles, particle size, the MDR strain or pathogen tested, Minimum Inhibitory Concentration (MIC), and a concise summary of the study findings.

**Table 1:** List of different nanocarriers used for the antimicrobial activity (*in vitro*/*in vivo* studies)^a^

S. no.	Nanocarrier	Type of nanoparticles	Size (nm)	MDR/pathogen	MIC value/effective conc	Finding/mechanism/conclusion	Reference
1	Metallic based	AgNPs	7	*E. coli*	3.38 μg mL^−1^	Damage DNA and disturb the synthesis of protein	164
				*S. aureus*	6.75 μg mL^−1^		
		MgO NPs	10.28	*C. albicans*	15.62 μg mL^−1^	ROS liberation, the interaction of MgO NPs with bacterial cell	165
				*K. pneumoniae*	15.62 μg mL^−1^		
				*E. faecalis*	7.8125 μg mL^−1^		
		Magnetic iron oxide nanoparticles	50–110	*E. coli*	DMF solution with 40 and 60 mJ laser fluencies	Could be due to stress generated by ROS disrupting bacterial cell membrane	166
				*P. aeruginosa*			
				*S. marcescens*			
		Sm-SeNPs (−)	170.6	*P. aeruginosa*	128 μg mL^−1^	Bacterial biofilm formation inhibition, disaggregation of the mature exo-polysaccharide matrix produced by microbes	167
		Bm-SeNPs (+)	160.6				
		Ch-SeNPs	102.5				
		Chitosan NPs	40	*E. coli*	0.25 μg mL^−1^	Chelation with trace elements that inhibit growth. Formation of an impermeable layer around the cell that prevents the transportation of essential solution	168
		Copper NPs		*S. choleraesuis*			
				*S. typhimurium*			
				*S. aureus*			
		AgNPs	3	*E. coli*	40–180 for AgNPs and 20–280 μg mL^−1^ for CuNPs	Mechanism is not clearly shown	169
		Cu NPs	10	*B. subtilis*			
				*S. aureus*			
		CuO NPs	20	*S. aureus*	10–100 μg mL^−1^	Copper ions from nanoparticle may be bind to negatively charged bacterial surface and can damaged cell wall, ultimately leads to cell death	170
				*B. subtilis*			
				*P. aeruginosa*			
				*E. coli*			
		AuNPs	52–55	*S. aureus*	10 μg mL^−1^	AuNPs generated holes in the bacterial cell wall, inhibiting the production of the peptidoglycan layer and increasing permeability and cellular leakage	171
				*E. coli*	100 μg mL^−1^		
		ZnO NPs	30	*C. jejuni*	0.05–0.025 mg mL^−1^	Disruption of the cell membrane and oxidative stress in *C. jejuni*	172
		AgNPs	23.17	*E. coli*	0.060–2.5 mg mL^−1^	Biosynthesized AgNPs possess antimicrobial activity against MDR strains	173
				*S.* spp.			
				*P. aeruginosa*			
				*A.* spp.			
				*C.tropicalis*			
		CuO NS	29.88	*S. aureus*	41.27 μg mL^−1^	Possible mechanism could be bacterial protein denaturation	94
				*E. aerogene*	48.38 μg mL^−1^		
2	Lipid based	LipoN	125	*L. monocytogenes*	320 IU mL^−1^	Enhanced antimicrobial activity could be due to diffusion process after fusing into the cell membrane	174
				*S. aureus*	640 IU mL^−1^		
		Liposome	77–86	*L. monocytogens*	5 log CFU mL^−1^ after 10 h for *L. monocytogens*	Nisin and lysozyme, maintaining their antimicrobial properties and offering stability and regulated release	175
				*S. Enteritidis*			
		Vancomycin loaded liposomes	100–200	Methicillin-susceptible and resistant	0.78 to 1.56 μg mL	Synthesized liposome could be reducing the formation and viability of mature biofilm	176
				*S. aureus*			
		MOX liposomes	100–150	*S. epidermidis*	0.09156 μM	—	177
3	Protein/polymeric based	Ciprofloxacin loaded chitosan NPs	72	*E. coli*	177 μg mL^−1^	Increased penetration of the drug into the bacterial cells, growth inhibition	178
					277 μg mL^−1^ without drug		
				*S. aureus*			
		Pluronic® F127 loaded with geraniol	26–412	*S. typhimurium*	MIC lower compared to geraniol	Synthesized nanoparticles lowered the concentration of geraniol required for inhibition of pathogen growth	138
				*E. coli*			
		CUR-NPs	200–300	*S. mutans*	260 μM	When curcumin and light interact in the presence of oxygen, reactive species are produced. So, this is might be reason which encouraged cell damage and death	140
				*C. albicans*			
				*MRSA*			
4	Micelles	Cationic micelles	—	*E. coli*	1.70–0.93 μM	Prepared cationic micelles specifically target the negatively charged cell membrane of *E. coli*. Once inside, the membrane is disrupted, allowing cytoplasm to flow out and ultimately causing cell death	179
		Cams	50–100	*S. aureus*	0.95 μg mL^−1^ for Gram-positive and 3.9 μg mL^−1^ for Gram-negative bacteria	The existence of an extra lipopolysaccharide layer, forming a hydrophilic barrier that keeps hydrophobic CAms from penetrating the membrane, may be the cause of higher MIC values of Gram-negative bacteria	180
				*L. monocytogenes*			
				*E. coli*			
				*S. typhimurium*			
				*P. aeruginosa*			
5	Dendrimer	Aspirin-based organoiron dendrimers	—	*B. subtills*	15 mg mL^−1^	Antimicrobial activity was increased against all the strains for the four complexes by increasing the dendrimer generation	181
				*S. aureus*			
				*E. coli*			
				*K. pneumoniae*			
		Vancomycin-PAMAM	—	*E. coli*	2, 2, 4 and 64 times decline compared to free vancomycin	Vancomycin can decrease the minimum inhibitory concentration (MIC) of Gram-negative bacteria by up to 64 times when G3 and G5 cationic PAMAM dendrimers are present. This may be due to increased vancomycin penetration through the bacterial membrane	134
				*K. pneumonia*			
				*S. typhimurium*			
				*P. aeruginosa*			
6	MSNs	C-TPA/nMC-48	—	*E. coli*	25 μg mL^−1^	Due to an increase in the penetrability of the cell membrane, E. Coli bacteria's thin peptidoglycan layer and cytoplasmic membrane could be ruptured in the presence of antimicrobial drug loaded MSN which cause cell lysis and leads to death	182
		Q-TPA/nMCM-48					
7	Hydrogel	ABA triblock copolymer	—	*E. coli*	99.8% killing efficiency with 1 wt% polymer	Successfully stop cell adhesion and suppress *E. Coli* growth through catechol-mediated hydrogen bonding and aromatic interactions inspired by mussels	183
		Hydrogel containing CuNPs	7	*E. coli*	1 to 5 mg mL^−1^	The interaction between released copper nanoparticles and Cu^2+^ ions and bacterial cell membranes produced the antibiotic activity, which interfered with biological processes and eventually damaged cells	184
				*K. pnemoniae*			
				*P. aeruginosa*			
				*P. vulgaris*			
				*S. aureus*			
				*P. mirabilis*			

a Abbreviations: MgO NPs: magnesium oxide nanoparticles, AgNPs: Sm-SeNPs (−): selenium nanoparticles synthesized by Gram-negative *Stenotrophomonas maltophilia*, Bm-SeNPs (+): selenium nanoparticles synthesized by Gram-positive *Bacillus mycoides*, Ch-SeNPs: synthetic selenium nanoparticles, AgNPs: silver nanoparticles, CuO NPs or Cu NPs: copper oxide nanoparticles, ZnO NPs: zinc oxide nanoparticles, CuO NS: copper oxide nanosheets, LipoN: nisin-loaded liposome, MOX liposomes: moxifloxacin (MOX)-loaded liposomes, CUR-NPs: curcumin loaded polymeric nanoparticles, Cams: cationic amphiphiles, MSNs: mesoporous silica nanoparticles, CLM loaded MSNs-NH2, clarithromycin loaded amine functionalized mesoporous silica nanoparticles, C-TPA/nMC-48: curcumin loaded 12-tungstophosphoric acid functionalized mesoporous silica nanoparticles, Q-TPA/nMC-48: quercetin loaded 12-tungstophosphoric acid functionalized mesoporous silica nanoparticles, ABA triblock copolymer: catechol functionalized polyethylene glycol (PEG) as A block and poly{[2-(methacryloyloxy)-ethyl] trimethylammonium iodide}(PMETA) as B block.

## 4 Challenges and future directions

Metallic nanostructures, lipid nanocarriers, polymeric nanocarriers, mesoporous nanomaterials, and nanofibers have been explored to tackle AMR; however, they possess some challenges and limitations. Biocompatibility and immunogenicity remain challenging, particularly with metallic nanoparticles, in the development of these nanocarriers.^185^ Optimizing drug-loading capacity is crucial, particularly in addressing drug-resistant infections where increased dosage may be required. Factors such as size, shape, and surface functionalization play a critical role, as they directly impact drug loading efficiency and the interaction of nanomaterials with biological systems.^186^ Striking the balance between enhancing antimicrobial efficacy and minimizing potential side effects is a key challenge. Additionally, maintaining stability during storage and manufacturing processes is essential, with particular care needed to preserve the integrity of nanomaterials, like protein-based nanomedicines are highly sensitive to environmental conditions.^187^ Therefore, optimization efforts must ensure that therapeutic properties are retained throughout the entire lifecycle, from production to clinical application.^188^

The translation of *in vitro* results to *in vivo* and clinically approved applications poses a significant challenge, as not all *in vitro* findings directly correlate with clinical success; extensive optimization protocols are often necessary. Proper optimization of physicochemical parameters and biological studies is essential. Factors such as physiological barriers, rapid systemic clearance, and complex interactions with the immune system can lead to discrepancies in the *in vivo* success compared to *in vitro* studies.^189,190^ These factors necessitate careful validation and optimization to ensure successful *in vivo* application. Additionally, the cost of manufacturing advanced nanocarriers presents another challenge, as these processes are often resource-intensive and require stringent quality control measures. Balancing efficacy with cost-effectiveness is crucial to making these nanotechnologies viable for large-scale clinical use.

Looking toward the future, optimizing nanomaterial characteristics such as size, shape, and surface properties should be a primary focus on research efforts. By integrating expertise from diverse fields such as medicine, biology, chemistry, and nanoscience, we can develop effective nanomaterials that play a crucial role in combating microbial infections and AMR. This multidisciplinary approach enhances our ability to innovate and nano-engineered therapies for more effective treatment outcomes.

## 5 Conclusion

Microbial infections caused by bacteria, viruses, and fungi are increasingly common, and the rise of AMR presents significant challenges to public health. This review highlights the challenges posed by AMR, the mechanisms underlying AMR, strategies to address it, and the evolving landscape of nanotechnology solutions to combat this global health threat. The rise of drug-resistant pathogens, driven by the overuse and misuse of antibiotics, calls for innovative strategies to address this crisis. Nanomaterials emerge as a beacon of hope, providing multifaceted solutions to the complexities of AMR. Key findings from this review underscore the significance of nanotechnology in combating AMR, emphasizing its potential to enhance antimicrobial therapies when combined with nanomedicines for the treatment of infections caused by resistant pathogens.

This focused review on nanotechnology as a weapon against AMR, serves as a roadmap for scientists and researchers eager to leverage nanomaterials in the fight against AMR. By providing comprehensive insights into a diverse array of nanomaterials, including metals, lipids, and polymers, facilitate a deeper understanding of their potential in addressing this global health challenge. These discussions on the innovative development and advantages of nanomaterial-based antimicrobial strategies will empower the future of potential treatments against microbial infections and AMR.

## Data availability

No primary research results, software or code have been included and no new data were generated or analysed as part of this review.

## Author contribution

R. S.: conceptualization, writing – original draft, data curation, software, N. M.: writing – original draft, R. K.: writing – original draft, M. J.: writing – review & editing, A. P.: writing – review & editing, D. B.: writing – review & editing, supervision. D. K. S.: conceptualization, writing – review & editing.

## Conflicts of interest

The authors declare no competing interests.
